# Seed Dormancy and Preharvest Sprouting in Quinoa (*Chenopodium quinoa* Willd)

**DOI:** 10.3390/plants10030458

**Published:** 2021-02-28

**Authors:** Emma M. McGinty, Kevin M. Murphy, Amber L. Hauvermale

**Affiliations:** 1 The School of Biological Sciences, Washington State University, P.O. Box 644236, Pullman, WA 99164, USA; emma.mcginty@wsu.edu; 2 Department of Crop and Soil Science, Washington State University, Pullman, WA 99164, USA; kmurphy2@wsu.edu

**Keywords:** abscisic acid, desiccation sensitivity, gibberellin, hormone signaling, precocious germination, seed morphology

## Abstract

Quinoa (*Chenopodium quinoa* Willd.) is a culturally significant staple food source that has been grown for thousands of years in South America. Due to its natural drought and salinity tolerance, quinoa has emerged as an agronomically important crop for production in marginal soils, in highly variable climates, and as part of diverse crop rotations. Primary areas of quinoa research have focused on improving resistance to abiotic stresses and disease, improving yields, and increasing nutrition. However, an evolving issue impacting quinoa seed end-use quality is preharvest sprouting (PHS), which is when seeds with little to no dormancy experience a rain event prior to harvest and sprout on the panicle. Far less is understood about the mechanisms that regulate quinoa seed dormancy and seed viability. This review will cover topics including seed dormancy, orthodox and unorthodox dormancy programs, desiccation sensitivity, environmental and hormonal mechanisms that regulate seed dormancy, and breeding and non-breeding strategies for enhancing resistance to PHS in quinoa.

## 1. Introduction to Quinoa, Cultivars, Breeding Issues, and Preharvest Sprouting

Quinoa (*Chenopodium quinoa* Willd.) is a pseudocereal originating from the Andes Mountain Range in South America and is a culturally significant staple food source that has been grown for thousands of years [1,2]. Due to its natural drought and salinity tolerance, quinoa has emerged as a favorable crop for production in marginal soils and in highly variable climates [3]). Quinoa’s nutrient dense grain is also ideal for supporting human health in diverse global communities, and for this reason, it has agronomic significance in global economies. 

There are five distinct quinoa ecotypes that originated from five different localities in South America. The original localities are (1) the valley habitat ranging across Colombia, Ecuador, Peru, and Bolivia, and the ecotype from this region is often tolerant to downy mildew; (2) the altiplano habitat which is near Titicaca Lake on the border of Bolivia and Peru, and the ecotype from this region is tolerant of marginal environments and frost; (3) the salares habitat, ranging across the salt flats of Bolivia and Chile, and the ecotype from this region is tolerant to high salinity; (4) the sea-level habitat, ranging from low-altitude areas of southern and central Chile, and the ecotype from this region is high yielding; (5) the subtropical or yungas habitat, ranging from the low-altitude humid valleys of Bolivia, and this ecotype is known for its late-flowering genotypes [1,3,4,5]. Until recently, there was limited pedigree information for quinoa, making it more difficult to identify the first quinoa ecotypes cultivated to produce today’s modern varieties. However, phenotypic and genotypic clues indicate that modern varieties have little to no seed dormancy, are xerophobic (meaning seeds display desiccation intolerance), and adult plants are salt and drought tolerant. These phenotypes are likely the result, at least in part, of adaptation to the original environments in which the first varieties were cultivated [1,3]. With the recent sequencing of the quinoa genome, it is expected that genetic tools will help to accelerate major breeding efforts for quinoa improvement focused on abiotic stress resistance, yield, and end-use quality [6]. 

From a production perspective, traits associated with enhanced plant plasticity that help to mitigate abiotic stress are beneficial for integrating quinoa into diverse cropping systems. For this reason, many research efforts have focused on understanding the genetic mechanisms that regulate abiotic stress responses, leading to drought or salt tolerance in adult quinoa plants [3,7,8,9,10]. Additionally, from a management perspective, the weak seed dormancy or in some cases, the absence of dormancy observed in quinoa is a desirable characteristic for integration into diverse crop rotations. This is because seeds that germinate readily are less likely to establish stable volunteer seed banks, requiring little to minimal input for management [11]. However, lack of seed dormancy has led to issues with reduced yields due to premature germination prior to harvest and has revealed a critical gap in knowledge about the regulation of seed dormancy in quinoa. Therefore, this review will define seed dormancy and the hormones involved in regulation, the different seed dormancy programs, the differences between orthodox and unorthodox dormancy programs, desiccation sensitivity, environmental mechanisms that regulate seed dormancy, and strategies for enhancing resistance against preharvest sprouting (PHS) in quinoa.

## 2. Model Systems: A Theoretical Framework for Quinoa Seed Dormancy, Hormone Signaling, and PHS

Seed dormancy is defined as a state in which seeds fail to germinate after receiving favorable environmental cues [12,13]. Dormancy classification is based on several factors including the developmental state of the embryo at the time of seed dispersal, physical characteristics of the seed, and physiological responses of seeds to environmental stimuli [14]. Historically, primary and secondary dormancy are most often used to describe differences in dormancy types for diverse plant species. Primary dormancy is characterized as dormancy induced on the mother plant during embryo maturation. Unlike primary dormancy, secondary dormancy is caused by the environment rather than inherited from the mother plant and occurs after seed maturation [15]. In addition to primary and secondary dormancy, other studies have revealed five additional subcategories of seed dormancy including physiological, morphological, morphophysiological, physical and combinational dormancy (Table 1) [14,15,16,17,18]. These categories, which may also influence primary and secondary dormancy, illustrate the nuanced and complex nature of dormancy. Seed dormancy and dormancy release are not binary processes but instead are regulated by a complex network of molecular, temporal, and physical cues, which ultimately result in germination [19,20]. The same is undoubtedly true for dormancy regulation in quinoa.

Primary dormancy is regulated by the plant hormone abscisic acid (ABA) [19,20]. As a seed transitions from dormancy to germination changes in physiology are largely controlled by the plant hormones, ABA, and gibberellin (GA) [21,22]. The hormone balance theory suggests that after seed maturation, as ABA signaling decreases there is a corresponding increase in GA signaling that leads to germination [23,24,25]. Many dormancy studies using model systems and cereals have established a clear connection between ABA and GA signaling with seed dormancy and dormancy loss and provide a theoretical framework for dormancy regulation in quinoa. Specifically, these studies have demonstrated the following: (1) higher seed dormancy is associated with higher endogenous ABA levels, and increased gene expression of ABA biosynthesis genes 9-cis-epoxycarotenoid dioxygenase 1 and 2 (*NCED1* and *NCED2*), (2) ABA levels and/or sensitivity decline during dormancy loss with a corresponding increase in sensitivity to GA, (3) at physiological maturity, a lack of seed germination in dormant seeds is associated with GA-insensitivity, and germination in nondormant seeds is stimulated by GA, (4) as dormant seeds after-ripen, dormancy is lost in stages reflected by changes in sensitivity to ABA and GA, and (5) with after-ripening seed dormancy loss occurs with decreased ABA hormone levels due to an increased expression of ABA catabolic genes ABA8′-hydroxylase 1 and 2 (*ABA8′OH1* and *ABA8′OH2*) and increased GA signaling [20,23,26,27,28,29,30,31,32,33,34,35,36,37,38,39,40,41,42,43]. Similar investigations evaluating dormancy release have demonstrated the following: (1) after-ripening decreases ABA sensitivity and increases GA sensitivity through increased GA biosynthesis and hormone accumulation resulting from GA20-oxidase gene expression, (2) decreased expression of GA2-oxidase, a GA catabolism gene, occurs as dormancy declines, (3) the GA GID1 (GA-INSENSITIVE DWARF1) hormone receptor increases with after-ripening and (4) as dormancy is lost ABA hormone accumulation decreases through increased ABA catabolism [19,20,21,23,37,38,43,44,45,46].

Seed dormancy studies in the Amaranthaceae family, that of which quinoa belongs, suggest that different quinoa varieties proceed through a combination of primary and physiological dormancy, or they have no dormancy (Table 1) [15]. Furthermore, close weedy relative of quinoa, *Chenopodium album* (common lambsquarter), and *Chenopodium berlandieri* were previously described as having primary dormancy [47,48,49]. Although these studies provide important phenotypic clues about the underlying mechanisms of quinoa seed maturation, dormancy, and germination in a broad sense, they fail to evaluate directly if there are different types of seed dormancy across quinoa varieties. For these reasons, more efforts are needed to implement a unified platform for characterizing and cataloging differences in seed dormancy phenotypes across varieties in a similar fashion as was done to link agronomic characteristics with regional ecotype.

**Figure 1:**
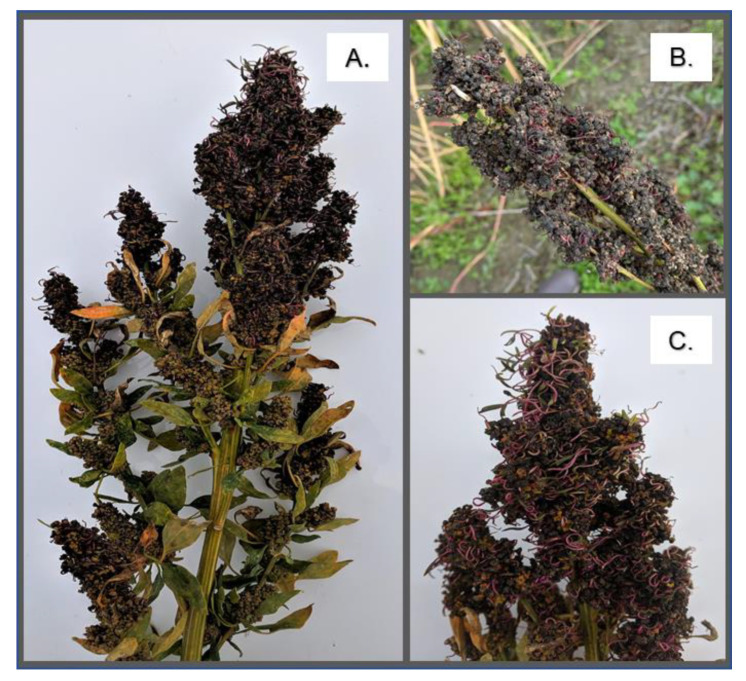
Quinoa preharvest sprouting (PHS). Panels A-C show inflorescences severely impacted by PHS in breeding line ‘3964′ from the 2015 and 2019 field seasons. Plants from the 2015 field season (**A**,**C**) were grown in Quilcene, WA on Dharma Ridge Farm. Plants from the 2019 field season (**B**) were grown in Skagit Valley, WA.

PHS is characterized by the germination of mature seeds on the mother plant due to rain or moisture prior to harvest (Figure 1) [50,51]. PHS is most often described through the lens of primary dormancy loss in model species and crops. Domestication and selective breeding for synchronized seedling emergence and stand establishment in many crops has resulted in decreased primary seed dormancy; seed dormancy mechanisms have been tailored to ensure maximum crop performance within the confines of a specific growing season [50,51]. PHS which results from altered primary dormancy is also known to occur through decreased ABA signaling and increased GA signaling [50]. The shift in the balance of hormone signaling leads to an extended seed germination window that is no longer synchronous with harvest [50,51,52]. It is important to note that both primary dormancy loss and the absence of seed dormancy increase the likelihood of PHS if rain occurs prior to harvest. However, the two physiological states are not synonymous and are likely regulated at the molecular level in different ways. Moreover, dormancy and PHS are complex traits that are regulated by multiple genes [50,51]. Therefore, to understand why quinoa is susceptible to PHS future studies in quinoa will need to evaluate whether PHS susceptibility is due to disruptions that change the architecture of primary dormancy and ABA and GA signaling or because quinoa seeds lack primary dormancy altogether.

## 3. Orthodox and Unorthodox Seed Types

**Figure 2:**
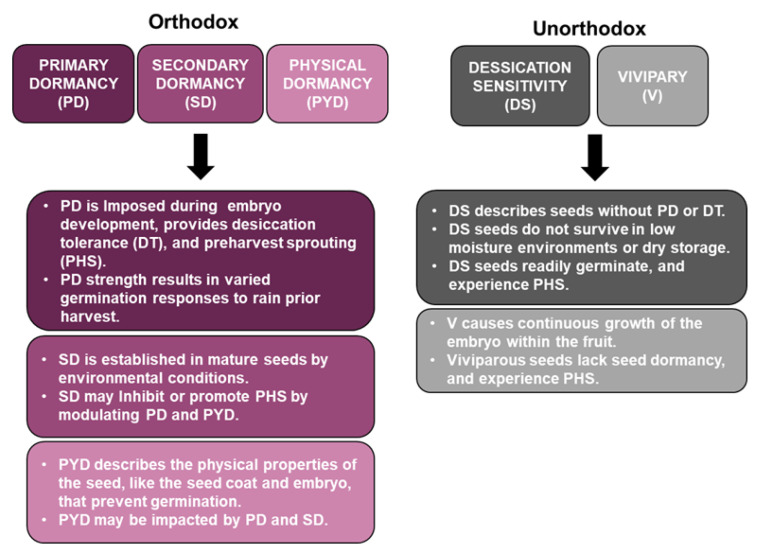
A summary of dormancy types associated with orthodox verses unorthodox seed types. Orthodox seeds display primary dormancy (PD), secondary dormancy (SD), and physical dormancy (PYD). Unorthodox seeds display desiccation sensitivity (DS) and vivipary (V). Specific dormancy programs and changes to these programs have implications for PHS susceptibility or resistance in quinoa.

Seed dormancy is an evolutionary adaptation that ensures the species survival of natural catastrophes within a specific environment [38]. Dormancy type is also associated with seed type and is different for orthodox versus unorthodox seeds (Figure 2). Orthodox seeds often display primary dormancy, which is marked by six chronological phases of development, including the following: (1) embryo growth and differentiation; (2) seed expansion, reserve storage, and vacuole filling; (3) internal desiccation, organellar de-differentiation, and membrane stabilization; (4) metabolic quiescence; (5) imbibition, reserve mobilization, and resumption of metabolic responses to environmental cues; and (6) germination [52]. Orthodox seeds are desiccation tolerant (DT) remaining viable for long periods of time in low moisture conditions [52]. Environmental cues such as light, temperature, and moisture not only impact the depth of primary dormancy, but they also play an important role in modulating secondary dormancy characteristics and the length of time required for complete dormancy release [38]. 

Seeds that lack one of the six previously described developmental stages associated with primary dormancy are classified as unorthodox seeds [52]. In many plant species, and weeds, unorthodox or discontinuous dormancy ensures germination only in favorable conditions, and it confers environmental plasticity, or the ability to respond to changing biotic or abiotic environmental factors [53]. Desiccation sensitivity (DS) otherwise known as recalcitrance, and vivipary, are two characteristics of unorthodox seeds [52]. Neither DS nor viviparous seeds display primary dormancy and both lack the third step in development necessary for desiccation tolerance. As a result neither seed type survives in low-moisture environments, or through periods of dry storage or freezing [52]. DS seeds are often from tropical environments, and if there is adequate soil moisture, DS seeds germinate immediately after dispersion [52]. Rather than persist in the seedbank similar to DT/orthodox seeds, DS/unorthodox seeds that do not germinate immediately after dispersion die. 

Elizabeth Farnsworth first suggested that quinoa produced “recalcitrant” or unorthodox seeds [52]. This characterization is based on the observation that many quinoa varieties that germinate at physiological maturity in wet environments, do not appear to survive as seeds in the soil, or they form stable seed banks. However, other quinoa germination studies have demonstrated that dormancy type and seed characteristics vary depending on quinoa variety, with some behaving as orthodox seeds with primary dormancy, while others do not [15,52]. It has also been hypothesized that seed desiccation status, such as primary and secondary dormancy, is the by-product of a plant’s natural environment and selection pressure, which is directly tied to the maternal line [54,55]. Additionally, most studies report that quinoa seeds lose their viability in a short time, especially in conditions of high humidity and temperature [56]. Poor seed viability has been largely attributed to poor storage conditions and a lack of uniform storage conditions. Poor seed viability is itself characteristic of many unorthodox seeds and suggests the possibility that desiccation tolerance or insensitivity in quinoa has largely been under-characterized and is largely not understood. Some research has suggested that there is a correlation between desiccation sensitivity, the generation of reactive oxygen species (ROS), and the occurrence of oxidative damage during dehydration in the seed [57,58]. Furthermore, this research suggests that desiccation tolerance depends on the seeds ability to scavenge ROS compounds by antioxidant defense systems [22,57,59]. Interestingly, heat and drought resistance in adult quinoa plants is thought to occur, at least in part, through the mitigation of ROS, and it is associated with increased peroxisome proliferation [3]. If ROS scavenging pathways are also involved in the desiccation status of quinoa seeds, i.e., tolerant or susceptible, then glyoxysome proliferation may be an important indicator for selecting DT quinoa varieties. 

Viviparous seeds are another group of unorthodox seeds, and they are common in monocot plant families such as Iridaceae (iris family) and Asparagaceae (asparagus family) [58]. Mangroves and corn are also two well-known examples of plants with viviparous seeds [60]. Similar to DS seeds, viviparous seeds have no primary dormancy, have a short viability window, and cannot survive dry storage or freezing [52]. Viviparous seeds also display PHS, although not all seeds that display PHS are viviparous [22,52,56]. Often in the literature, vivipary is used as a synonym for PHS due to the phenotypic similarities between both. However, the molecular architecture of the two seed physiologies is quite different, and to date there have been no studies to evaluate if quinoa PHS results from vivipary, lack of seed dormancy or both [52]. Simple germination screens have routinely been deployed to evaluate characteristic changes in hormone sensitivity associated with primary dormancy and dormancy loss, as well as the underlying causes of PHS physiology in many plant species. Thus, germination screening platforms will be essential for elucidating underlying mechanisms regulating PHS physiology across diverse quinoa germplasm.

## 4. Physical Dormancy in Quinoa

Physical dormancy is an additional subcategory of orthodox dormancy, involving the embryo, seed coat, or both [61,62,63]. Embryo dormancy is characterized by an external or internal physical or biochemical block that prevents embryo growth and germination [63]. Seed coat-imposed dormancy often occurs in seeds with a hard, impermeable shell that requires physical perforation or damage to germinate [63]. In some populations, it is possible that both embryo and seed coat-imposed dormancy play a role in quinoa germination programs and need to be carefully described to evaluate if and how each contributes to PHS. 

In a recent study, seed coat thickness was measured in two varieties of quinoa, Chadmo and 2-Want, to evaluate if differences in observed dormancy occurred because of seed coat-imposed dormancy and in turn impacted PHS [64]. 2-Want had a thinner seed coat, while the Chadmo had a thicker seed coat. When the seed coats were perforated, both varieties continued to display a basal level of dormancy. Hormone analysis determined that seed coat thickness negatively correlated with the amount of endogenous ABA leached from the seed during development. Varieties with thinner seed coats leached more ABA and were less dormant than varieties with thicker seed coats. However, in both cases seed coat disruption did not completely alleviate dormancy. This finding suggested that in addition to seed coat-imposed dormancy, some quinoa varieties also have embryo dormancy [64]. This result is important because it suggests that at least in some quinoa varieties, vivipary is not a contributing factor in PHS sensitivity.

In addition to seed coat thickness, seed coat color has also been implicated in the regulation of quinoa seed dormancy and may be associated with ABA signaling mechanisms. The connection between seed coat color and dormancy regulation has been documented in other plant species including cereals such as wheat and barley, which are members of the Amaranthaceae family [48,49,65,66]. Comparisons between red versus white wheats indicate that red seed coat color is associated with stronger seed dormancy [50,67]. Additionally, a study evaluating the role of seed coat color in wheat found a quantitative trait locus (QTL) for PHS in the same location as coat color and suggested that coat color is likely to play an important role in PHS sensitivity or tolerance [68]. Interestingly, studies evaluating the link between seed coat color and dormancy depth in close relatives of quinoa, C. *album*, C. *berlandieri*, and C. *bonus-henricus*, observed that darker seed coat color was associated with stronger dormancy [48,61]. Furthermore, studies exploring differences in dormancy associated with heteromorphic populations of *C. album* found that brown seeds had thinner seed coats, and no primary dormancy. Whereas, black-colored seeds had thicker, and sometimes stronger seed coats, which are thought to act additively to enhance primary dormancy [49]. Likewise, seed coat studies evaluating dormancy in quinoa varieties such as Chadmo and Titicaca indicated that dormancy is stronger in seeds with a darker colored coat [64]. Another study, evaluating the *Suaeda salsa* in Amaranthaceae, found similar results [47]. Brown seeds had higher germination rates and absorbed water better than black seeds [47]. However, a key limitation of these studies is that they only compare dormancy associated with very dark (black or brown) and light seed coats (light brown) and do not evaluate the broader range of seed coat colors that exist in quinoa including those that are yellow, red or pink [69,70,71,72,73]. Therefore, future studies will need to examine the genetic connection between seed coat thickness, color, and ABA signaling mechanisms, as well as associated impacts on dormancy programs across a wide collection of quinoa cultivars. 

## 5. Environmental Regulation of Quinoa Seed Dormancy

Secondary dormancy is regulated by the environment and is temporally separated from primary dormancy [38]. Environmental factors reported to enhance secondary dormancy in many species, including quinoa, are photoperiod or day length, temperature, precipitation, and altitude [2,3,6,12,71,72,73,74,75]. Environmental factors that influence the depth of secondary dormancy often do so through increased ABA signaling. Interestingly, previous studies investigating abiotic stress responses, specifically those connected to drought and salinity tolerance in adult quinoa plants also proceed through ABA signaling networks [76,77]. However, there is no research that has investigated whether the ABA signaling networks that contribute to abiotic stress tolerance in adult quinoa plants are also involved in modulating PHS physiology during seed germination. However, what is known is that as the photoperiod increases endogenous, ABA and sugar levels increase in quinoa seeds. Increases in endogenous ABA and sugars are important indicators of embryo dormancy and “ripening” [76,77,78]. Based on these findings, it was concluded that ABA and sugar signaling are possible mechanisms that regulate the photo-adaptability of adult quinoa cultivars [77]. Long photoperiods may also promote stronger dormancy by increasing ABA levels in seeds [52,78,79,80,81,82]. 

In addition to photoperiod, temperature plays a role in quinoa development, and it likely impacts seed dormancy and germination. In a study addressing how the environment can affect quinoa, two varieties Chadmo and 2-Want, were exposed to different temperatures and photoperiods to gauge germination and dormancy capacity [78]. The authors discovered that high temperatures and long photoperiod days increased dormancy. It was also determined that the temperature window has increased for quinoa, meaning that modern quinoa varieties have adapted to germinate in colder temperatures than their earlier relatives, and that growing environment is the biggest factor impacting seed dormancy [78].

The cultivation of quinoa across diverse regions, including in hot and dry climates, is likely to have contributed to the requirement for higher germination temperatures. However, understanding the relationship between germination rates and temperature is confounded by the fact that quinoa can germinate in a wide array of varying temperatures. One study reported maximum germination rates at 30 °C [78]. However, other studies have found that the optimum germination temperature is 37 °C [80]. A shift in recent breeding strategies to cultivate quinoa across very diverse environments has likely had a significant impact on plant and seed physiology [76]. The result of changes to breeding practices and environments has resulted in varieties with low dormancy but high adaptability [78]. These results suggest that growth and germination temperatures may have different effects on distinct varieties of quinoa. These results also suggest that these effects may be exacerbated by environments and may have major implications for variations in seed dormancy phenotypes depending on local growing conditions. 

Precipitation and altitude also influence quinoa seed dormancy. Its widely known that quinoa is tolerant to drought and mildew, and these desirable characteristics are a direct result of the original growing habitats [3,54]. It is also known that rainfall prior to harvest when seeds are mature may result in PHS. The five original ecotypes come from very diverse environments, with precipitation varying from intense wet mountains to dry sandy regions [3,4,5,54]. It is important to mention this, because to date, although there have been some studies that have tested how different quinoa varieties respond to varied precipitation and drought treatments, none have directly investigated how precipitation timing near harvest maturity impacts PHS susceptibility.

Seed coat thickness and color, two factors that impact dormancy strength, may also be regulated by the environment. For example, a study evaluating the effect of elevation on seed coat thickness and rates of germination for quinoa’s close relative *C. bonus-henricus* found that seeds grown at a lower elevation had thinner seed coats, and increased rates of germination [61]. If seed dormancy mechanisms are conserved in quinoa then it might be expected that varieties grown at higher elevations will have thicker seeds coats, slower rates of germination, and perhaps more seed dormancy than those grown at lower elevations. It is also good to note while looking at color differences, that seeds with thinner seed coats appear to be lighter in color, whereas thicker coats have darker coloration [64].

## 6. Breeding Strategies to Mitigate PHS in Quinoa

Research studies in cereals such as wheat and barley, and model plants like *Arabidopsis thaliana* has demonstrated a compelling connection between increased seed dormancy and PHS resistance [3,12,45,52,54,78,83,84]. In the cases of wheat and Arabidopsis, several major and minor quantitative trait loci (QTLs) associated with increased PHS resistance map to regions of the genome containing genes previously characterized as regulators of dormancy and seed coat color [50,68,85,86]. Therefore, breeding for stronger seed dormancy in quinoa seems a promising approach for reducing the risk of crop losses due to PHS. Two strategies currently being used to accomplish this task are 1) to make crosses between quinoa varieties displaying different levels of seed dormancy, and 2) to make crosses between quinoa and wild relatives, such as native lambsquarter (*C. berlandieri*) which is a tetraploid similar to quinoa and has more clearly defined seed dormancy. The primary goals of both approaches have been to create hybrid populations that allow for a better understanding of how seed dormancy and PHS are segregating within a population, and to create PHS resistant germplasm. 

In the first strategy, breeders have used Titicaca, a cultivar developed in Demark with higher seed dormancy, and ‘Chadmo, QQ065-PI 614880′, which is a naturally dormant variety originating from the Chiloe island in Chile, to create quinoa populations with increased seed dormancy and PHS resistance [3,64,78,82]. However, despite these efforts, incidences of PHS in both Titicaca and Chadmo have been reported with adequate rainfall prior to harvest, and across diverse growing regions (breeder listening sessions; International Quinoa Conference, 2020). Additionally, hybrid populations created at the Sustainable Seed Systems Laboratory at Washington State University using Chadmo also displayed frequent PHS in the higher rainfall zones of Western WA, despite initially appearing to be resistant (K. Murphy, personal communication). Taken together these results suggest that while many quinoa varieties appear to be susceptible to PHS, with some displaying some form of seed dormancy, there is not a clear connection between the type or level of seed dormancy with level of PHS susceptibility. These results also suggest that the mechanisms of regulation between dormancy and PHS in quinoa may not be analogous to those in cereals or other model plant species. However, with the recent sequencing of the quinoa genome, many genomics-assisted breeding approaches, including QTL analysis, and the molecular characterization of PHS-specific genes, are now possible [6,50]. 

The second approach developed to increase PHS resistance in quinoa is to introduce the desired dormancy type and level, i.e., primary and strong, by making a wide cross with a genetically compatible relative. The objective of this approach is to add desirable traits that are currently lacking in the existing genetic pool. Selective breeding approaches have been routinely implemented in other crops, using landraces or weedy relatives to increase physiological plasticity to abiotic stresses, and to increase disease resistance [87].

Although classified as an invasive weed species in the United States, *Chenopodium berlandieri,* also known as pitseed goosefoot, is grown as a seed crop in other parts of the world. *C. berlandieri* has emerged as a possible candidate for increasing PHS resistance in quinoa because (1) it is genetically compatible with quinoa and a cross between the two produces viable offspring, and (2) it displays strong orthodox primary seed dormancy, and this dormancy diminishes in a trackable manner over time that would also allow for simultaneous PHS sensitivity screening. Pitseed goosefoot is comprised of two subspecies, *berlandieri* and *nuttaliae*, and ecotypes can be found growing from southern Mexico and Texas, into southwestern and eastern North America, including along the coasts of the Atlantic and Gulf of Mexico [88]. Since pitseed goosefoot was a source of food for centuries in pre-European indigenous cultures in eastern North America, it is adapted to regions of the U.S. where quinoa struggles to grow. Pitseed goosefoot is a potential donor parent of key agronomic traits in quinoa, including heat tolerance, nutritional value, and resistance to PHS.

However, it is important to mention the possible risks associated with this approach, namely the development of germplasm that will establish a robust volunteer seedbank. From an agronomic perspective, two of the most favorable characteristics of existing quinoa varieties are that they germinate readily and do not survive desiccation, meaning they do not establish weedy seed banks. Hybrid populations generated from wide crosses will serve as essential tools for characterizing dormancy physiology and PHS regulation in quinoa. If used for variety development, future breeding efforts using wide crosses will need to strike a balance between increased seed dormancy and decreased germinability, so as not to trade PHS susceptibility for volunteer seedbank establishment. 

In addition to the previously mentioned strategies aimed at increasing PHS resistance through increasing quinoa seed dormancy, another approach is to select for faster maturing varieties. The advantages to this “avoidance strategy” is that neither dormancy status nor PHS sensitivity are factors if harvest time is separated fall rains. However, the challenge with this approach, however, is that it does not address quality issues associated with dormancy, or PHS physiology, relying instead on optimum growing conditions and weather stability. In an age of increasing climate variability, it is unclear how current quinoa production regions might be impacted or how future production systems will need to be tailored to target optimal growing regions. 

## 7. Other Tools for Mitigating PHS in Quinoa

Breeding for increased resistance to PHS in quinoa is a long-term endeavor taking years to produce new varieties. Given that very little information is understood about quinoa seed dormancy structure and PHS physiology, breeding strategies that rely heavily on the conventional wisdom established for PHS in cereals may be confounded by differences in biological and environmental factors that regulate seed maturation, germination, and viability in pseudocereals. Therefore, future research should also incorporate non-breeding, short and intermediate-term PHS mitigation strategies that help alleviate the risk of PHS in quinoa. Quinoa seed physiology may fall outside of an orthodox dormancy regime, with varieties displaying weak dormancy to no primary dormancy, DS, as well as a significant decline in seed viability post-harvest [89]. For these reasons, in addition to trying to increase PHS resistance through breeding for increased seed dormancy, it might also be necessary to minimize PHS risks by transiently modulating seed physiology from DS to DT in the field just prior to harvest. 

One way to temporarily change seed physiology is by using growth regulators. Paclobutrazol (PAC) is a GA biosynthesis inhibitor historically used to understand the dynamics of GA and ABA signaling networks in dormant and germinating seeds [90]. Previous studies evaluating PAC treatments in quinoa have done so in adult plants and have focused on increasing drought and salt tolerance as well as yield [3,48,83]. In all cases PAC treatments were efficacious for increasing resistance to abiotic stress and for increasing yields and did so through an indirect increase in ABA signaling resulting from decreased GA signaling. Additional work investing the regulation of DS in the seeds of *Citrus limons,* a species without seed dormancy, found that treating freshly harvested seeds with PAC both slowed the rate of seed germination and extended the seed viability window from weeks to months, which are two phenotypes consistent with orthodox seed dormancy [52].

Expression analysis comparing non-treated and PAC-treated seeds demonstrated that in addition to inhibiting GA biosynthesis and signaling genes, PAC treatment also inhibited other phytohormone pathways associated with cell growth, including auxin and ethylene signaling, as well as causing an increase in both endogenous ABA hormone levels and signaling. Furthermore, studies evaluating the efficacy of ABA treatments for mitigating DS in the seeds of *Acer Saccharinum* indicated that ABA alone was able to rescue a DS phenotype through increased ABA-mediated signaling [90]. Analysis of total protein in ABA-treated seeds found that ABA-treated seeds accumulated higher amounts of Late Embryogenesis Associated (LEA) proteins which are associated with seed dormancy and desiccation tolerance [90]. Although PAC is a powerful research tool for understanding dormancy regulation, due to issues with toxicity, it is not suitable for large-scale production systems. However, unlike PAC, ABA is routinely and safely used to stimulate fruit ripening and enhance fruit color in grape production systems [91]. In a similar way, timed ABA treatments just before seed physiological maturity may prove to be powerful tool for enhancing PHS tolerance and increasing seed viability in quinoa through a transient increase in dormancy. 

## 8. Conclusions

Over the last decade quinoa has emerged as a high-value, nutritious crop to enhance food security; it is artisanal (domestic interest), and it performs well in variable environments and in marginal soils. For these reasons, many research efforts have centered on understanding and improving traits related to abiotic stress tolerance, disease resistance, and yield. In many cases studies have focused on adult plants. Consequently, one largely overlooked area of research has been the study of the mechanisms that regulate quinoa seed dormancy. In the last few growing seasons, across the globe from Rwanda to the Pacific Northwest of the U.S.A., diverse quinoa varieties have been plagued by PHS, including those previously bred for enhanced dormancy. In some instances, untimely rains before harvest have resulted in nearly complete crop losses due to PHS. Therefore, the overall goal of this review paper is to inform breeders and non-breeders alike about the complex physiology leading to PHS in quinoa. 

The strategies we believed to be key for understanding the dynamic nature of quinoa seed dormancy and PHS physiology include (1) the development of a high-throughput hormone screening pipeline, to quickly characterize the presence or absence of dormancy, and baseline dormancy strength at physiological maturity, (2) the implementation of a PHS screening platform modeled after those routinely used to evaluate PHS susceptibility in wheat, (3) the development of gene-specific primers to evaluate changes in genes associated with ABA and GA signaling, seed dormancy, PHS tolerance, and desiccation sensitivity/tolerance, and (4) assessing the effects of ABA and PAC treatments on dormancy preservation, seed germination rates, and desiccation tolerance in greenhouse and field trails. These efforts will provide a framework for developing new tools for understanding seed physiology in quinoa and mitigating PHS.

## Figures and Tables

**Table 1:** A summary of recognized seed dormancy categories [15].

Dormancy Category	Description
Primary	Established during embryo maturation by the plant hormone abscisic acid (ABA) and prevent germination.
Secondary	Established in mature seeds by environment stimuli preventing germination.
Physiological	Physiological responses to environmental or hormonal stimuli that prevent germination.
Morphological	Fully differentiated embryos remain physically too small to carry out radicle/cotyledon emergence.
Morphophysiological	Physiological responses and physical limitations that prevent germination.
Physical	Specialized physical features of the seed that prevent germination.
Combinational	Specialized physical features and physiological responses that prevent germination.
Embryo	External or internal physical or biochemical signals that prevent embryo growth and germination.
Seed Coat-imposed	Imposed by a hard, impermeable seed coat requiring physical damage to induce germination.
